# Determinants of stillbirth among women deliveries at Amhara region, Ethiopia

**DOI:** 10.1186/s12884-017-1573-4

**Published:** 2017-11-13

**Authors:** Demeke Lakew, Dereje Tesfaye, Haile Mekonnen

**Affiliations:** Statistics Department, Science College, Bahir Dar University, P.O. Box: 79, Main Campus-Peda, Bahir Dar, Ethiopia

**Keywords:** Stillbirth, Logistic regression, Bi-variable, Multi-variable, Amhara region

## Abstract

**Background:**

Stillbirth is one of general medical issues that could contribute significantly to creating nations like Ethiopia. This study aimed to determine the prevalence and related factors of stillbirth among deliveries at Amhara region, Ethiopia.

**Methods:**

The study used the Ethiopian Mini Demographic and Health Survey (EMDHS) data collected from 2555 eligible Amhara region women in 2014. Bi-variable and multi-variable binary logistic regression analysis was used.

**Results:**

The prevalence of stillbirth outcomes became 85 per 1000 (total live birth). Besides, majority of women did not attend any formal education and had no antenatal care follow up. Women whose age at first birth below 18 years were 1859(72.8%) and the mean preceding birth interval were 33.6 months. Even women who attended primary and above education were about 50% and they were less likely to have had stillbirth outcomes than those who had no education (AOR: 0.505, 95% CI 0.311–0.820) and women having higher household wealth index were less likely to have had stillbirth outcomes as it is compared to the reference category. Moreover, women having preceding birth interval above 36 months were about 89% of less likely to end up stillbirth outcomes as compared to women having preceding birth interval below 24 months (AOR: 0.109, 95% CI 0.071–8.0.168).

**Conclusions:**

It could be inferred that a stillbirth result is one of the general medical issues in Amhara Region. Among different factors considered in this study, age, age at first birth, wealth index, birth order number and preceding birth interval in months were found to be significantly associated factors for stillbirth. Therefore, more awareness of early birth, widening birth interval, enhancing maternal care (for aged women) and early birth order number could be recommended.

## Background

Pregnancy loss is a collective term used to describe pregnancies that failed to produce a live birth. The losses can occur anytime during the course of pregnancy. A stillbirth is characterized as the demise of the embryo in the uterus before birth at or following 28 weeks’ incubation. Intrauterine demise can happen either earlier or amid work [1]. Following the development in neonatal intensive care, the definition has changed and varies between countries. In high-income countries, like Norway, a newborn can survive after 25 weeks’ gestation, compared to 28–32 weeks’ gestation in low resource countries. In many low resource countries, preterm babies with no life expectancy outside the womb, therefore die intrauterine without any attempt at rescue.

The stillbirth rate for developed nations is predicted between 4.2 and 6.8per thousand births, whereas for the developing countries, it is estimated as 20 to 32 per thousand births. Sixty-six percent of all stillbirths occur in only regions such as Africa and South-East Asia [2]. In sub-Saharan Africa, an expected amount, 900,000 pregnancy experienced stillbirth. Stillbirths occurrence varies greatly among countries [3]. For example, countries with less knowledge on preventing stillbirths, give stillbirths low national priority [4]. In Ethiopia, the world health statistics 2013 showed a stillbirth rate of 26 per thousand deliveries that makes it the third maximum rate in the east African nations next to Djibouti and Somalia (with stillbirth costs of 34 and 30 per 1000 births, respectively) [5]. The Ethiopian Demographic and Health Survey (DHS) 2005 data showed that the still birth rate is 18 for each 1000 births [6].

Every year, nearly 3 million third trimester stillbirths happen globally with 98% in low and middle-income countries (LMIC) [7]. Interventions to reduce stillbirths are not often evaluated, and those studies of enhancing maternal and new child fitness have rarely covered the evaluation of their effect on stillbirth. Though, stillbirth is not recognized as a fitness problem, either in worldwide health metrics or in specific context, most LMIC data report the stillbirth cases [1]. Yet stillbirths are invisible in international fitness monitoring. Unlike other unfavorable pregnancy outcomes (including maternal and neonatal mortality), stillbirth is not always formally included in any of the most important global sickness campaigns [4]. That is, if a pregnancy final result is not counted, it will almost definitely be ignored by the investment companies, policy makers, and neighborhood communities. There is also a giant fatalism that those toddlers who die of utero have never been intended to stay. Even the stigmatization of ladies who have given birth to a dead toddler is unfair, merciless and not based on facts. Besides, the point fatalism concerning stillbirths among care givers and policy makers will actually guarantee that no development happens. Hence, fitness professionals, and lots of ladies for whom they offer care consider stillbirth a good deal more than is typically perceived, but the topic is not often discussed in the media, or by means of policy makers [8]. In addition, stillbirths are regularly no longer registered systematically in lots of low-income countries like Ethiopia, and reliable registrations exist only in countries with minor quantity of deaths. This leads to underestimation of stillbirths in those countries, where 98% of all stillbirths occur [1].

In general, the invisibility of stillbirths is complicated and deeply rooted in social constructs of personhood in high mortality settings including Amhara region, Ethiopia. For example, blame and stigma, in addition to the prevalence of home delivery and the shortage of a national vital registration system, contribute to the invisibility of stillbirths. Acknowledging stillbirth while it occurs requires each nearby and country wide reaction, along with the development of a national vital registration system, health system policies, and behavior-change communication interventions.

Based on the aforementioned rationale, this study aimed to determine the prevalence and associated factors of stillbirth outcomes of pregnancy.

## Methods

### Study design and area

The 2014 Mini Ethiopia Demographic and Health Survey (EMDHS) were carried out by the Central Statistical Agency (CSA) with the aegis of the Ethiopian Ministry of Health. The EMDHS used a stratified sample selected in two stages from the Population and Housing Census (PHC) frame. The stratification was carried out by way of selecting each place as urban and rural. Thus, nationally representative sample of 22,036 eligible women aged 15–49 were interviewed, of which, 2555 were from Amhara region [9]. The Amhara region is located in the North-Western and North Central parts of Ethiopia and situated within 90 and 23,045′N and 360 and 40,030′E. The region’s elevation ranges from 700 m, in the Eastern parts, to over 4620 m in the North West. The region also, has a total area of 170,000km^2^ and is divided into 11 administrative Zones and 105 districts.

### Data collection

The survey was designed to produce representative estimates for the country as a whole, for the urban and the rural areas one at a time, and for each of the 11 regions. The EMDHS used a household and woman’s questionnaire to gather information on background characteristics, birth history and youth mortality, knowledge and utilization of family planning methods and antenatal, delivery and postnatal care. These instruments adopted from the 2011 Ethiopia DHS based on the requirements of clients of the EMDHS [9]. Data quality was guaranteed by using a pre-tested data collection tool and trained data collectors. Staff from the CSA was in charge of managing the everyday technical operations including enlistment and training of field and data handling staff and the supervision of the workplace and field operations.

### Measurement

The response variable for this study, stillbirth outcomes (1 = stillbirth occurred and 0 = does not occur) was characterized as the introduction of a newborn child that has passed on in the womb or amid intra partum following 28 weeks of growth. The predictor variables were socio-demographic and economic characteristics (residence, age, educational level, wealth index and age at first birth) and obstetrics related characteristics (number of ANC Visits, time of first ANC visit and preceding birth interval in months and others). The wealth index was also computed, national-level wealth index/quintiles by Ethiopian Statistical Agency from poorest to richest utilizing family unit resource data by means of a principal components analysis, and in this study, this was categorized as poor, middle and rich [9]. Besides, preceding birth interval in months was categorized as below 24 months, between 25 and 36 months and above 36 months.

### Ethics statement

The national survey was led by the standards communicated in the Declaration of Central Statistical Agency (DCSA), Ethiopia. The survey was also approved by the Ethical Review Board (ERB) at CSA and all individuals who agreed to participate in the survey signed a consent form.

### Data analysis

Data were entered into SPSS version 21 for analysis by CSA data clerk team. The permission was granted by the CSA, Ethiopia to use the data for this article. Descriptive statistics like frequencies/ percentages and cross tabulations were used to evaluate normality, outliers and perceived lacking values. Then, multi covariate logistic regression was carried out by way of getting into all variables with a *P-*value less than 0.25 in the bivariate analysis. Finally, binary logistic regression with forward likelihood selection method having a *P-*value less than 0.05 was used to identify the determinant factors associated with stillbirth outcomes. The binary logistic regression is the sort of regression that is used whilst the dependent variable is a dichotomous and the independent variables are of any kind. This model estimates the probability that a characteristic is presented as given the values of explanatory variables:

$$ \log it\left(\frac{P(event)}{1-P(event)}\right)={\beta}_0+{\beta}_1{X}_1+{\beta}_2{X}_2+{\beta}_3{X}_3+\dots +{\beta}_k{X}_k. $$

The quantity to the left of the equal sign is the log of the odds that an event takes place. Such methods allow researchers to rank the relative significance of independent variables assess interaction effects, and to understand the impact of covariate control variables. The crude and adjusted Odds Ratio (OR) and its 95% CI were estimated.

## Results

In this study, a total of 2555 women were involved. The results of this study are presented in three tables. Table 1 shows the demographic characteristics. Table 2 states the obstetrics related characteristics and Table 3 has medical related characteristics.

**Table 1:** Socio-demographic characteristics of sampled women, Amhara Region Ethiopia

Characteristics	Frequency (*n* = 2555)	Percent
Residence		
Urban	164	6.4
Rural	2391	93.6
Age		
< 24	178	7.0
25–34	903	35.3
35+	1474	57.7
Marital Status		
Married	2184	85.5
Other^a^	371	14.5
Educational Level		
No education	2131	83.4
Primary and Above	424	16.6
Wealth Index		
Poor	1254	49.1
Middle	752	29.4
Rich	549	21.5
Age at First Birth		
Below 18	1859	72.8
Above 18	696	27.2
Family Size		
Below 5	1072	42.0
Above 5	1483	58.0
Birth Order Number		
First	604	23.6
Second	510	20.0
Third and Above	1441	56.4

^*a*^Majority is divorced

**Table 2:** Obstetrics related characteristics of sampled women in Amhara Region Ethiopia

Characteristics	Frequency	Valid Percent
Did you attend antenatal care?		
No	2313	90.5
Yes	242	9.5
Number of Antenatal care Visits		
No Visit	147	38.1
1+	239	61.9
Time of first Antenatal Care visit		
1st trimester	58	24.5
2nd trimester	124	52.3
3rd trimester	55	23.2
Modern Contraceptive Used Prior to Current Pregnancy		
No	1621	63.4
Yes	934	36.6
Type of Contraceptive Used		
Not Using	1594	62.4
Injections	758	29.7
Other^b^	203	7.9
Place of Delivery		
Home	465	88.4
Health Center	61	11.6
Preceding Birth Interval in Months		
< 24	897	46.0
25–36	540	27.7
36+	512	26.3

^*b*^majority are Implant/Norplant

**Table 3:** Medical related characteristics of sampled women in Amhara Region Ethiopia

Characteristics	Frequency	Percent
Stillbirth Outcomes		
No	2337	91.5
Yes	218	8.5
Signs of pregnancy complications (vaginal bleeding and gush of fluid, severe head ache, blurred vision, fever, abdominal pain, swollen legs, other)		
No	2515	98.4
Yes^b^	40	1.6
Blood Pressure		
No	80	33.1
Yes	162	66.9
During pregnancy, given or bought iron tablets/syrup		
No	230	59.0
Yes	160	41.0

^*b*^Majority is abdominal pain

As Table 1 shows, only 6% of women aged 15–49 were from urban areas and more than half (58%) were above 35 years old. More than three fourth of the women (83.4%) had no formal education and only 22% of the women aged 15–49 were in the rich wealth quintile.

Based on Table 2, one in every ten women of 15 to 49 years old, in Amhara region, (10%) made one or extra antenatal visits during the route of pregnancy. Of these women 42% were using modern contraceptive methods. Besides, the most popular contemporary contraceptive method utilized by 30% of the women, 15 to 49 years old, was injectables.

As Table 3 shows, some 218 (8.5%) women had stillbirth outcomes. Only 40 (1.6%) women had signs of pregnancy complications. Of which abdominal pain experienced the majority, and considerable number of women, 160 (41%) were taking iron. On top of these pregnancy complications, about 162 (66.9%) women were diagnosis hypertensive as their systolic and diastolic blood stress with greater than 120 mmHg and 80 mmHg based on American Heart Association (AHA) classification.

### Multivariate analysis for predicting stillbirth outcomes

One problem of single covariate approach is that it ignores the opportunity that a collection of variables, each of which is weakly associated with the final results, and this can turn out to be a vital predictor of the outcome whilst taken collectively. It is miles consequently essential to reduce the possibility of excluding variables on the bivariable analysis level. It is for this reason that a bi-variable test *p*-value of 0.25 or much less used for selection of variables for the multi covariate analysis from single covariate findings [10]. Totally based on such lists like residence, age, educational level, wealth index, antenatal care attendance, number of antenatal care visits, modern contraceptive used, kind of contraceptive used, place of delivery and preceding birth interval in months were selected for forward likelihood method.

As Table 4 indicates, after adjusting other covariates, women whose age below 24 years was about 71% less likely to have stillbirth outcomes than those whose age was above 35 years old (AOR: 0.288, 95% CI 0.126–0.656). Similarly, women aged between 25 and 34 years was about 78% less likely to have had stillbirth outcomes than those whose age was above 35 years old (AOR: 0.221, 95% CI 0.155–0.314). The women attended primary and above education was about 50% less likely to have had stillbirth outcomes than those of having no education (AOR: 0.505, 95% CI 0.311–0.820). In addition, women having wealth index rich and the middle was about 70% (AOR: 0.302, 95% CI 0.204–0.447) and 71% (AOR: 0.290, 95% CI 0.213–0.396) less likely to have had stillbirth outcomes than those of having poor wealth index.

**Table 4:** Logistic regression analysis of factors associated with stillbirth in Amhara Region, Ethiopia

Variables	Stillbirth		*P*-Value	Crude OR (95% CI)	*P*-Value	AOR^*^ (95% CI)
	No	Yes				
Residence						
Urban	153	11	0.000	0.072 (0.039–0.133)		
Rural	2184	207		1		
Age			0.000		0.000	
< 24	159	19	0.000	0.119 (0.074–0.192)	0.003	0.288 (0.126–0.656)
25–34	839	64	0.000	0.076 (0.059–0.098)	0.000	0.221 (0.155–0.314)
35+	1339	135		1		1
Educational Level						
No education	1950	181		1		1
Primary & Above	387	37		0.096 (0.068–0.134)	0.006	0.505(0.311–0.820)
Wealth Index			0.000		0.000	
Poor	1155	99		1		1
Middle	682	70	0.000	0.103 (0.080–0.131)	0.000	0.290 (0.213–0.396)
Rich	500	49	0.000	0.098 (0.073–0.131)	0.000	0.302 (0.204–0.447)
Did you attend antenatal care?						
No	2101	211		1		
Yes	236	7	0.000	0.030 (0.014–0.063)		
Number of Antenatal care Visits						
No Visit	138	9		1		
1+	231	7	0.000	0.030 (0.014–0.064)		
Modern Contraceptive Used Prior to Current Pregnancy						
No	1483	138		1		
Yes	854	80	0.000	0.094 (0.074–0.118)		
Type of Contraceptive used			0.000			
Not Using	1458	136		1		
Injections	687	71	0.000	0.103 (0.081–0.132		
Others	192	11	0.000	0.057 (0.031–0.105)		
Place of Delivery						
Home	443	22		1		
Health Center	52	6	0.000	0.115 (0.050–0.269)		
Preceding Birth Interval in Months					0.000	
< 24	793	104				1
25–36	520	20	0.000	0.038 (0.025–0.060)	0.001	0.093 (0.059–0.148
36+	489	23	0.000	0.047 (0.031–0.071)	0.0001	0.109 (.071–0.168

*Keys*: *COR* (Crude odds ratio), *AOR* (Adjusted Odds Ratio), *CI* (Confidence Interval), 1 (Reference Category), ^*^Variables(s) entered in step 1 is wealth index, in step 2 is preceding birth interval, in step 3 is age and in step 4 is educational level

The preceding birth interval was the strongest predictor of stillbirth outcomes. Women having preceding birth interval above 36 months were about 89% less likely to end up stillbirth outcomes as compared to women having preceding birth interval below 24 months (AOR: 0.109, 95% CI 0.071–8.0.168). Likewise, women having a preceding birth interval between 25 and 36 months were about 91% less likely to end up stillbirth outcomes as compared to women having preceding birth interval below 24 months (AOR: 0.093, 95% CI 0.059–0.148).

## Discussion

In this study, prevalence and related factors of stillbirth results at Amhara regional state, Ethiopia was assessed and the prevalence rate of stillbirth was 85 per 1000 total births. This prevalence is higher than a study conducted in four low and middle-income countries and this showed a stillbirth rate of 30 per thousand deliveries [11]. This number is also higher than the previous reports of the world health statistics 2013 stated as a stillbirth rate of 26 per 1000 in Ethiopia, 34 per 1000 in Djibouti and 30 per 1000 births in Somalia [5]. Moreover, as the 2005 EDHS Survey [6] data showed stillbirth rate was 18 per 1000 births [6]. This is because the consecutive Ethiopian DHS was underreported because of omission and missed reporting [6, 12–14]. Underreporting occurs for some of the motives, such as inclusive of the exercise of keeping apart women and their newborns within the early postnatal length, and reputation of new child loss of life as regular, and a perception that the new child is not someone for a detailed time frame [6]. Besides, additionally almost all stillbirths were underreported and the underreporting of stillbirth was disregarded in the international health schedule [7, 12, 15, 16]. These things could happen because now these days stillbirths are often now not registered systematically in lots of low-income countries which need p in underestimation of stillbirths in these nations, wherein 98% of all stillbirths occur as well as reliable registrations exist only in countries with a minor number of deaths [7]. In addition, stillbirths in Ethiopia in general and in Amhara region in particular are attributed to supernatural powers which do not need to be talked about then they are buried within the house or inside the outdoor [12]. This prevalence is even higher than what Gondar University hospital reported as 71 per 1000 total births [17]. Such may be because giving birth at the hospital had better facilities than those who did difference home. In this study, the majority of the women (88.4%) gave birth at home.

A woman in a rural home will meet several challenges if an acute obstetric emergency occurs. Limited finances and no available transportation were two major obstacles as poor women had given higher stillbirth outcomes than that of rich women [17, 18]. The Ethiopian government has been trying to provide community-based emergency transportation (ambulance) though the existing road next works do not create access to rural women in Amhara region, Ethiopia. Among the socioeconomic status wealth index was also associated with stillbirth outcomes in that women who had higher wealth index experienced fewer stillbirth outcomes compared to those who had low wealth index. This finding is consistent with Ha et al. study which reported that as women in the poorest two quintiles they have had more threat of stillbirth as compared to the richest women [19].

Moreover, in this study showed that older maternal age is associated with higher stillbirth rates. Compared with women of 35 years old, there was about 71% increment of the risk in stillbirth for women less than 24 years old [20], and about 78% increment of risk in stillbirth for women 25 to 34 years old. The result of this study also similar to Audu et al. study in Nigeria [21] which showed women less than 35 years old, the stillbirth rate is increased two-fold for women with 35 to 39 years old, and added three to four-fold for women aged 40 or above. Consequently, older women stillbirth threat rises more rapidly as gestational age increases due to some age-associated threat had higher rates of maternal complications.

In bivariable analysis, women who had more than one ANC followed up during the current pregnancy were about 97% less likely to have a stillbirth than women did now not have ANC follow up.

This finding could support the previous studies that used is in keeping with preceding studies at Gondar hospital using multivariate analysis at Gondar Hospital [17]. For example, Akilew et al. stated that regular ANC follow up could help a pregnant woman seek early treatment for her potential pregnancy associated troubles and have access to information related to nutrition and hazard signs and symptoms of being pregnant. On the other hand, the researchers noted that women who did not have longer ANC follow up may not be benefited from basic ANC packages.

This study farther showed women who had above 36 months preceding birth interval were about 89% less likely to have had stillbirth outcomes than those of women their preceding birth interval was below 24 months. Such result is in congruent with the findings of a study by Elizabeth et al. as they suggested that short inter-being pregnant intervals are a few of the maximum common threat factors for stillbirths in developing nations [22]. In addition, Assefa et al. stated that the threat of pregnancy loss changed into notably reduced for women who had a pregnancy interval of two or extra years in comparison with women who had a being pregnant interval of below two years [23].

## Conclusions

Authors concluded that stillbirth is one of the public health problems in Amhara Region, Ethiopia. Among different factors considered in this study, age, age at first birth, wealth index, birth order number and preceding birth interval in months were significantly associated factors for stillbirth outcomes. Moreover, older maternal age and short preceding birth interval were associated factors for stillbirth.

Hence, further awareness of early birth and wider birth interval are important recommendations. Authors also recommend further enhancements of maternal care for aged women as well as during early birth order number.

## Abbreviations

ANC: Ante natal care
AOR: Adjusted odds ratio
CI: Confidence interval
COR: Crude odds ratio
LMIC: Low and middle income countries
OR: Odds ratio
SD: Standard deviation
SPSS: Statistical packages for social Sciences
